# Consensus Conference on Clinical Management of pediatric Atopic Dermatitis

**DOI:** 10.1186/s13052-016-0229-8

**Published:** 2016-03-02

**Authors:** Elena Galli, Iria Neri, Giampaolo Ricci, Ermanno Baldo, Maurizio Barone, Anna Belloni Fortina, Roberto Bernardini, Irene Berti, Carlo Caffarelli, Elisabetta Calamelli, Lucetta Capra, Rossella Carello, Francesca Cipriani, Pasquale Comberiati, Andrea Diociaiuti, Maya El Hachem, Elena Fontana, Michaela Gruber, Ellen Haddock, Nunzia Maiello, Paolo Meglio, Annalisa Patrizi, Diego Peroni, Dorella Scarponi, Ingrid Wielander, Lawrence F. Eichenfield

**Affiliations:** Pediatric Allergy Unit, Research Center, San Pietro Hospital - Fatebenefratelli, Rome, Italy; Dermatology, Department of Experimental, Diagnostic and Specialty Medicine, University of Bologna, Bologna, Italy; Pediatric Unit - Department of Medical and Surgical Sciences, S. Orsola Malpighi Hospital, University of Bologna, Pad. 16, Via Massarenti, 11 – 40138 Bologna, Italy; Pediatric Department, “S. Maria del Carmine” Hospital of Rovereto, APSS (Provincial Agency for Health Services), Trento, Italy; Institute of Relational Psychotherapy – IPR, Rome, Italy; Pediatric Dermatology Unit, Department of Medicine, University of Padua, Padua, Italy; Pediatric Unit, San Giuseppe Hospital, Empoli, Italy; Institute for Maternal and Child Health, IRCCS Burlo Garofolo, Trieste, Italy; Pediatric Unit, Department of Clinical and Experimental Medicine, University of Parma, Parma, Italy; Department of Medical Sciences, Section of Paediatrics, University of Ferrara, Ferrara, Italy; Pediatric Clinic, University of Verona, Verona, Italy; Dermatology Unit, Bambino Gesù Children’s Hospital, IRCCS, Rome, Italy; Department of Pediatrics, Central Hospital of Bolzano, Bolzano, Italy; Departments of Pediatrics and Dermatology, School of Medicine, University of California, San Diego, CA USA; Department of Woman, Child and General and Specialized Surgery, Second University of Naples, Naples, Italy; Primary Care Pediatrician, Health National Service, Rome, Italy

**Keywords:** Atopic dermatitis, Childhood, Consensus, Management

## Abstract

The Italian Consensus Conference on clinical management of atopic dermatitis in children reflects the best and most recent scientific evidence, with the aim to provide specialists with a useful tool for managing this common, but complex clinical condition. Thanks to the contribution of experts in the field and members of the Italian Society of Pediatric Allergology and Immunology (SIAIP) and the Italian Society of Pediatric Dermatology (SIDerP), this Consensus statement integrates the basic principles of the most recent guidelines for the management of atopic dermatitis to facilitate a practical approach to the disease. The therapeutical approach should be adapted to the clinical severity and requires a tailored strategy to ensure good compliance by children and their parents. In this Consensus, levels and models of intervention are also enriched by the Italian experience to facilitate a practical approach to the disease.

## Background

Utilizing the best and most recent scientific evidence, the aim of this Consensus is to provide specialists with a useful tool for managing this apparently simple, but in reality complex, clinical condition. To this purpose, the Consensus has been divided into three main sections (Topical therapies, Systemic therapies, Non-pharmacological interventions), and the key aspects of each of these have been considered. Summary boxes in the text highlight the fundamental measures to be implemented, with details delineated within each section. The important topic of therapeutic education is discussed within section 3. The selection of therapies for individuals with atopic dermatitis should be influenced by the clinical appearance of lesions, and the application of topical products requires a tailored approach to ensure good compliance by children and their parents. In this Consensus, levels and models of intervention are based on the Italian experience, but also informed by models of care in other countries such as Germany, where therapeutic education for atopic dermatitis is approved and reimbursed by the National Health System. The basic principles of the “International Dermatology and Allergy Guidelines for Atopic Dermatitis Management” are integrated into this consensus statement to facilitate a practical approach to the disease [1].

**Fig. 1:**
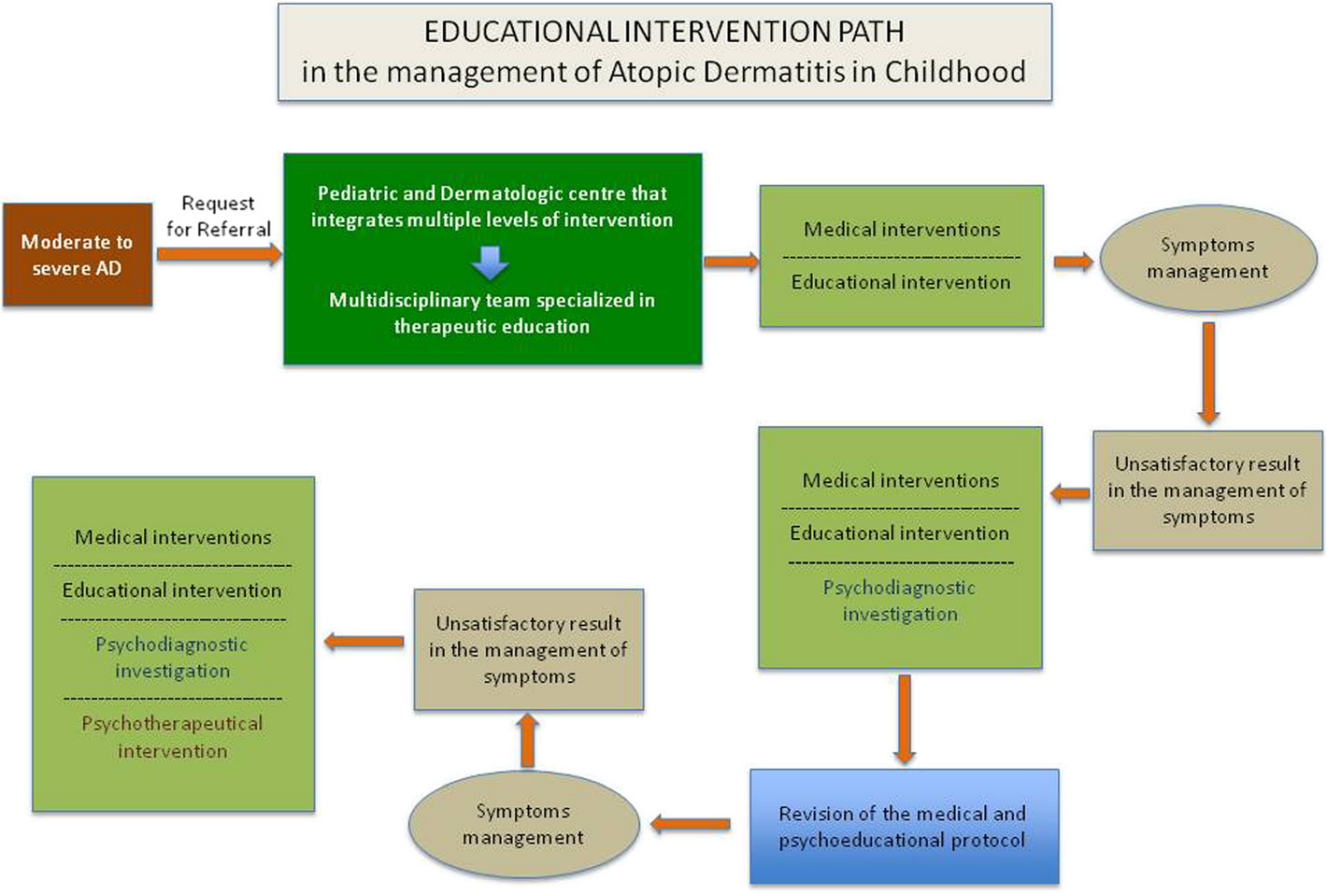
Patient educational program for cases of moderate to severe AD

## Topical therapies

### Skin care: moisturizers and cleansers

Maintaining skin barrier function is an important therapeutic goal and a priority of treatment algorithms from the most prominent scientific societies worldwide [2–6]. However, validated therapeutic regimens for acute and chronic phases of atopic dermatitis (AD) in children are still lacking. AD is known to be a chronic disease, which in most cases is mild or moderate but can be severe enough in some patients to require systemic therapy. The clinical picture is typically characterized by alternate periods of remissions and exacerbations, while it can also be persistent. With mild and moderate cases, a properly conducted topical therapeutic regimen is usually sufficient to obtain good control of the AD.

#### Hygiene: detergents

Cleansing is recommended for patients with AD as part of maintenance therapy (strength of recommendation C - level of evidence II, according to the recent US guidelines) [4]; however, there is no unanimity in recommendations about the frequency and duration of washes [4, 7]. In fact, the guidelines on the one hand suggest frequent washing to remove crusts and allergens (eg. dust mites) and to reduce colonization by *S. aureus*, while on the other warn against damaging the skin barrier with cleaning that is too aggressive [3–6]. Some authors suggest the use of daily baths with lukewarm water of short duration (eg. 5 min), followed by gentle drying with smooth cloths and application of emollients while the skin is still damp [8]. When inflamed lesions are poorly responsive to anti-inflammatory treatment, the so-called *soak and smear* method has been recommended because it can produce a higher absorption of topical steroids; this method consists of washing for 20 min followed by the application of topical anti-inflammatory steroid, without first drying the affected areas [9, 10]. Although there are currently few clinical studies, the use of non-soap cleansers and synthetic detergents (the so-called syndets) are recommended; these products have a slightly acidic pH (preferably about 5.5–6), should be free or relatively free of preservatives and perfumes, and have liquid formulations to facilitate rinsing [4, 11, 12]. Recently, the recommendation of the use of washes with diluted sodium hypochlorite (NaClO) has been reaffirmed; dilute NaClO was studied in 2007 and shown to be effective in countering the proliferation of S. aureus, a known cause of AD flares [3, 4, 8, 13, 14]. This treatment is recommended in combination with nasal mupirocin in patients with moderate-severe AD and clinical signs of bacterial superinfection [4] (see Chapter 1.3 “Topical antimicrobials”). To date, the addition of oils or other antiseptic products to detergents is supported by a few studies, but with conflicting results and without randomized controlled clinical trials (RCTs) they cannot be recommended for clinical practice [4, 15, 16]. Oily cleansers containing mineral oils are preferred to minimize allergenicity, but the short duration of contact with the detergent normally decreases its ability to induce sensitization and trigger contact eczema [16].

#### Moisturizers - emollients

The use of topical moisturizers and emollients is an essential element of the treatment and prevention of xerosis recommended by published guidelines (strength of recommendation A, level of evidence I) [2–5]. Randomized controlled clinical trials conducted on the use of topical moisturizers have proven their effectiveness in preventing AD relapses with a consequent reduction in the use of topical steroids [17–25]. Emollient products may be classified according to their composition (Table 1), with different proportions of emollient agents. These agents moisturize the skin and reduce transepidermal water loss through occlusive properties (eg. Vaseline), or by attracting and holding onto water through humectant properties (eg. collagen, hyaluronic acid and other molecules with high molecular weight) [4]. Preparations with a hydrophilic base (for example preparations containing urea in percentages varying from 5 to 10 %) are available and can be used, depending on the type and site of the lesions to be treated (especially in dry and very dry areas) and the age of the child and the age of the child (i.e. emollients containing urea above 3 years of age) [3]. Some of the more recently marketed emollients contain various molecules such as glycerol, analogues of the Natural Moisturizing Factor (NMF), and lactic acid and may act by improving hydration and the integrity of the skin barrier. In recent years other emollients that contain physiologic lipids (ceramides, polyunsaturated fatty acids and cholesterol, which promote epidermal differentiation and correct the deficiency of lipids among corneocytes) have been developed; when it’s possible the use of third generation emollients is preferable. It is not possible to give definitive guidance concerning the frequency of application and the amount of product to use, because there are not sufficient clinical studies to address these issues [4, 26]. However, recent guidelines suggest modulating the frequency of application according to xerosis, climatic conditions and patient sports/activities (eg. swimming) [24]. It is recommended to use a proper amount of product quantities of (150–200 g/week are recommended for children, and up to 500 g/week is recommended for adults) [3, 24]. Most of the currently available products are emulsions, more or less fluids, creams or milks. Products with a higher fat content may be preferred during the winter season [3]. Finally, there are medicated cosmetics and medical devices which are also proposed as therapeutic aids (prescription emollient devices; PEDS). They contain a base to which natural anti-inflammatory agents such as bisabolol (obtained from chamomile), glycyrrhetinic acid (obtained from liquorice), and/or essential omega 6 fatty acids are typically added. However, controlled studies comparing the effectiveness of these products are still limited and they have not demonstrated greater efficacy than traditional agents [6, 14, 21, 27–34] (Table 2). Recently, some articles have considered the use of specific emollients in the primary prevention of AD in genetically susceptible infants; a trial conducted on 124 infants at high risk for AD has shown that the application of an emollient on the skin surface reduces by 50 % the incidence of the disease evaluated at 6 months of life [35] and similar results were observed by other authors in preterm infants [36–38].

**Table 1:** Characteristics of the main emollients

	Product	Action
1st-generation emollients	Vaseline, paraffin oil, fatty alcohols, hydrophilic polymers (collagen, ac. hyaluronic acid, chitosan, polysaccharides gelling)	Hygroscopic and occlusive
2^nd^-generation emollients	Glycerol, sorbitol, substitutes NMF (Natural Moisturizing Factor) derivatives of pyrrolidone carboxylic acid, urea (5–10 %), lactic acid, ammonium lactate	Restoring hydration and barrier function
3^rd^-generation emollients	Physiological lipids: ceramides, cholesterol, polyunsaturated fatty acids	Barrier repair therapy

**Table 2:** Main studies conducted on the use of emollients in pediatric patients with atopic dermatitis (modified from Mack Correa MC. et al. 2012 [6])

Population	Treatment	Frequency and duration of application	Efficacy	Safety
Infants (age <12 months) with moderate to severe AD (*n* = 173). Grimalt et al. 2007 [21]	Emollient containing oat extract (Exomega, Laboratories Pierre Fabre, France)	Twice/day for 6 weeks	Significant reduction in the use of high potency topical CS and improvement of SCORAD and QoL	Two severe reactions.
				Good tolerability in 94 % of patients.
Infants and young children (aged 2 months-6 years) with mild to moderate AD (*n* = 25) Nebus et al. 2008 [30]	Occlusive cream containing colloidal oatmeal and detergent with colloidal oatmeal and glycerin (Aveeno, Johnson & Johnson Consumer Companies, Inc., Skillman, USA)	Cream: twice/day for 4 weeks	Significant improvement of IGA, dryness and itching at 2 and 4 weeks; QoL significantly improved at 4 weeks	Well tolerated; no severe reactions related to treatment
		Cleansing: every wash		
Children (aged 3 months-16 years) with mild to moderate AD (*n* = 65) Kircik et al. 2011 [31]	Emulsion containing ceramides (EpiCeram)	Twice/day for 3 weeks	Improvement of IGA, patient satisfaction and QoL	No severe reactions to the treatment
Children with AD (aged 6 months-12 years) (*n* = 76) Giordano-Labadie et al. J 2006 [32]	Moisturizing milk (Exomega) compared to control	Twice/day for 2 months	Significant improvement of dryness, itching and QoL	Satisfactory or excellent level of tolerance in 97 % of patients
Children (aged 6 months-12 years) with mild to moderate AD (*n* = 142) Breternitz et al. 2008 [17]	Glycyrrhetinic acid based cream (Atopiclair) compared to vehicle	Three times/day for 43 days	Significant improvement of IGA, reduced use of topical corticosteroids	No severe reactions related to the treatment
Children and adolescents (aged 6 months-18 years) with mild to moderate AD (*n* = 121) Sugarman et al. 2009 [29]	Emulsion-containing ceramides (EpiCeram) compared to topical fluticasone (Cutivate, Pharmaderm, Melville, NY, USA)	Twice/day for 28 days	Significant improvement in SCORAD index. Comparable effectiveness between the two treatments	No severe reactions related to treatment
Children (aged 1.5–12 years) with resistant treatments/recalcitrant AD (*n* = 24) Chamlin et al. 2002 [27]	Emulsion containing ceramides (Triceram, Osmotics Corp., Denver, CO, USA) instead of the previous moisturizer, continuing topical tacrolimus or topical corticosteroids	Twice/day for 12 weeks, then once/day for 9 weeks	Significant improvement of SCORAD in 92 % of patients within 3 weeks, in 100 % within 21 weeks; decrease of trans-epidermal water loss; hydration and integrity of the stratum corneum improved	No severe reactions related to treatment
Children and adolescents (aged 2–17 years) with mild to moderate AD (*n* = 39) Miller et al. 2011 [33]	Glycyrrhetinic acid based cream (Atopiclair) vs. ceramide- based emulsion (EpiCeram) vs. petrolatum-based ointment (Aquaphor Healing Ointment, Beiersdorf Inc., Wilton, CT, USA)	Three times/day for 3 weeks	Improvement in the 3 treatment arms with no difference; Ointment-based petrolatum showed the best improvement measured through clinical evaluation	No severe reactions related to treatment
Children and adults (aged 2–70 years) with mild to moderate AD (Study 1, *n* = 66; Study 2, *n* = 127) Simpson et al. 2011 [34]	Cetaphil Restoraderm moisturizing (Galderma Laboratories, Fort Worth, TX, USA)	Study 1: twice/day for 4 weeks; Study 2: twice/day for 4 weeks in addition to topical corticosteroid.	Study 1: significant decrease in pruritus and improvement of hydration and QoL. Study 2: only compared to steroid: significant improvement of hydration, decrease in EASI score and more rapid action	No severe reactions related to treatment

### Summary box

#### Cleansing

- Bathe for short amounts of time in warm water with gentle cleansers relatively free or free of preservatives and perfumes.
- Dry skin gently with smooth cloths
- In case of bacterial superinfection, use wash containing dilute sodium hypochlorite.

#### Hydration

- Emollients are the backbone of AD therapy.
- The most appropriate formulation should be chosen and applied at least daily to the entire surface of the skin in sufficient quantity to minimize xerosis, which may be affected by weather conditions and sports/activities.
- The constant use of emollients, even during remission, prevents flares and decreases the use of topical steroids.

### Topical corticosteroids

Topical corticosteroids (TCS) are still considered the mainstay of pharmacological treatment and the first choice drugs for AD therapy [4]. The use of TCS for eczema goes back about 60 years, and their effectiveness has been demonstrated in more than one hundred RCTs [39]. They are anti-inflammatory medications that can be applied to damaged skin to treat eczema in any stage of inflammation and to reduce itching [39–41]. They act on a multitude of cells of the immune system, such as T lymphocytes, monocytes, macrophages and dendritic cells. It is hypothesized that TCS may act by interfering with the mechanisms of antigen processing, by binding to specific cell receptors, and by suppressing the release of pro-inflammatory cytokines [4, 41]. Taking into account these mechanisms of action, it is proposed that the antipruritic effect of TCS is best appreciated when the itching is secondary to inflammation [41]. The optimal dose of TCS can be standardized in *finger tip units*, a quantity of product that can be squeezed from a tube along the distal phalanx of the index finger of an adult hand; this amount containing approximately 0.5 g is the proper amount to be applied to a surface as large as two palms of the hands of an adult [42]. TCS should be applied after adequate cleansing of the area to be treated [43].

In order to achieve long-term therapeutic effects, important consideration should be give to:

A) **Choice of TCS,** which depends on the strength and vehicle of the preparation. TCSs are categorized into four groups according to strength; with group I being the weakest and group IV being the strongest (Table 3). In addition, the vehicle affects the strength of TCS, with creams generally less strong than ointments containing same active drug component. The choice of the formulation depends on the surface area and location of eczema [44] (Table 4).

**Table 3:** Topical corticosteroids are divided into 4 groups according to their power (from Patrizi and Gurioli [270])

Group I	Group II	Group III	Group IV
Low power	Moderately powerful	Powerful	High power
• Hydrocortisone • Hydrocortisone Acetate	• Aclometasone dipropionate • Clobetasol butyrate • Dexamethasone sodium phosphate • Dexamethasone valerate • Desonide • Fluocortinbutilestere • Hydrocortisone butyrate	• Beclomethasone dipropionate • Betamethasone benzoate, dipropionate and budesonide valerate • Budesonide • Desossimetazone • Diflucortolone valerate • Diflucortolone valerianate • Fluocinolone acetonide • Fluocinonide • Fluocortolone • Fluocortolone caproate • Fluticasone propionate • Methylprednisolone aceponate • Mometasone furoate • Prednicarbate	• Halcinonide • Clobetasol propionate

**Table 4:** Choice of CTS formulation according to the phase and location of AD

Phase of eczema	Formulation of CTS
Dry erythema	Cream/Milk
Erythema with lichenification	Ointment
Erythema with exudation	Lotion/Cream
Hairy areas	Lotion/foam/gel

However, we must always keep in mind some considerations:

- TCS are indicated for the treatment of eczema and not of xerosis and/or lichenification; these manifestations should be treated with moisturizers after resolution of the acute phase;
- Ointments can cause folliculitis;
- Topical agents should be applied in a thin layer until absorbed, as excess material can cause irritation;
- The selected formulation should be cosmetically acceptable to the patient in order to encourage good compliance.

B) **Frequency of application and duration of treatment**. The use of TCS is most commonly once or twice a day, preferentially in the evening, as there is no evidence that more frequent application produces better results; indeed, there are no convincing data to suggest that daily administration is less effective than twice daily [45, 46]. There is no consensus in the literature regarding the duration of therapy. Medium-high potency TCS are generally preferred in the acute phases because they can lead to a significant benefit in the short term [47]. These therapies should be continued over each single lesion until its resolution of eczema is achieved, then can be reduced and gradually stopped or changed to a less strong TCS (according to the concept of minimum effective dose) [4]. Greater attention must be paid to thinner skin, eg. eyelids or neck, because there is a greater risk of absorption and local atrophy. In these locations, especially if frequent applications of TCS are needed, preparations with reduced systemic absorption and lower risk of skin atrophy are preferred. Examples include newer generation TCS (eg. fluticasone propionate, methylprednisolone aceponate, mometasone furoate, desonide) or immunomodulators such as topical calcineurin inhibitors (see Chapter 1.4 “Topical immunomodulators”).

Once the healing is achieved (regression of eczema and improvement or resolution of itching), the goal of therapy should be to extend the relapse-free time as long as possible. For this purpose, emollients can be effective, but patients or family members should be educated to reapply topical steroid immediately in case of relapse. The maximum monthly amount of medium or high potency TCS to be used to avoid local and/or systemic side effects is equal to 15 g in infants, 30 g in children, and 60–90 g in adults [3, 42]. In the last few years, another type of approach to maintanance care is being promoted, the so-called “proactive” approach. This new treatment strategy is particularly useful for optimizing clinical control in patients with frequent AD flare-ups. The “proactive” therapy should be started after eczema heals. It consists of applying TCS intermittently once or twice a week in the areas most prone to relapse, even when no inflammatory lesions are visible. This strategy reduces the number of relapses and lengthens the interval free from symptoms more effectively than the use of emollients alone [42, 48]. The rationale of proactive therapy is to obtain control of subclinical disease by using minimal amounts of TCS [46, 49]. In patients with exudative lesions and/or erosions, the application of TCS and even emollients can cause burning and may be poorly tolerated, especially in young children. In these cases the use of compresses may reduce exudation and promote re-epithelialization of lesions. The incidence of side effects from new generation TCS is very low [4]. However, the long-term use of TCS, especially if high potency, may cause local side effects. In rare cases, systemic effects may occur, more frequently in children due to the high ratio of total body surface area to body mass, which is about 2.5 to 3 times higher than for adults [50]. Normally, just 1 % of the TCS applied supplies all of its therapeutic action; the remaining 99 % is removed from the skin surface by rubbing, washing and exfoliation [50]. Despite this, prolonged exposure to high potency or older generation TCS, as may occur with occlusion, may lead to significant systemic absorption and suppression of the hypothalamic-pituitary-adrenal axis, especially if there is concurrent use of corticosteroids for other conditions (rhinitis, asthma) [51]. Main side effects of TCS are striae rubrae, skin atrophy, telangiectasia, skin burning, erythema and acneiform eruptions [52].

Among the side effects that are most feared is local skin atrophy [53]. The mechanism through which skin atrophy can be induced includes the inhibition of fibroblast proliferation, as well as reduced synthesis of collagen. Recent data are fairly reassuring about the risks of skin atrophy; in a study exploring skin thickness before and after prolonged use of moderate quantities of medium and high power topical steroids (not exceeding the above recommendations), atrophy was not observed in the treated areas [52]. Other studies have shown that skin atrophy due to TCS is a reversible phenomenon, and that healing may occur after a few weeks of TCS therapy discontinuation [54]. The possibility of contact dermatitis should be taken into consideration, especially when eczema worsens or does not respond to properly administered TCS therapy [4]; in these cases, the performance of patch tests for diagnostic purposes is indicated. Steroid-resistance may develop in case of superinfection with staphylococcus [55]. Even more rare is the observation of systemic side effects on growth, hyperglycemia, hypertension and glaucoma, reported in very old studies during systemic therapy [56] and even less frequently described for local therapies [50].

A systematic review of the literature on the side effects of TCS concluded that the safety profile of new TCS is good when used as directed [57]. However, corticophobia is commonly observed among families and even doctors [58, 59]. This is a condition to be avoided, as insufficient and inadequate applications of TCS limit ability to control dermatitis and can worsen therapeutic compliance [52, 60]. Especially in severe forms of AD, it is therefore important to implement a multidisciplinary approach, in which therapeutic education has an important role. TCSs constitute the first choice therapy for eczema, and in the majority of cases, they are able to maintain good control of the dermatitis, with benefits that greatly exceed the uncommon iatrogenic risks [50].

### Summary box

- TCSs represent the cornerstone therapy for eczema, especially for the moderate/severe forms: they should be applied on damaged, erythematous and/or exudative skin.
- Their effectiveness is related to their potency (Table 3), the vehicle of the preparation (Table 4), and the application modality.
- TCS potency and vehicle (ointments, creams, lotions, milks or foams) should be chosen depending on the age of the patient, sites and types of eczema.
- Once daily application in the evening and continued use until complete resolution of lesions is a preferred method
- In cases of relapsing AD, “proactive” therapy should be encouraged, with the application of TCS twice a week (in the evening) on areas of frequent relapses.
- The safe monthly dose of medium to high potency TCSs is 15 g in infants, 30 g in children, 60–90 g in adolescents-adults.
- Newer generation TCS have a good safety profile, especially if used properly.

### Topical antimicrobials

Skin infections in patients suffering from AD are most frequently caused by S. aureus, S. pyogenes and H. simplex virus (HSV). In patients with AD, the prevalence of colonization of the skin and/or nose by S. aureus varies from 60 to 100 %, while in control subjects without AD it varies from 5 to 30 %. A correlation between bacterial colonization and severity of eczema has also been reported in the literature [61]. Some toxins produced by S. aureus act as superantigens: they are able to produce a massive activation of T cells and contribute to exacerbation of skin lesions. The toxins also seem to induce the production of specific IgE, the activation of basophils, and consequently, the inflammatory cascade [62]. Topical antibiotic therapy is indicated for treatment of monofocal bacterial infections or confined impetigo, but not for simple colonization. Most topical antibiotics are available in two formulations: cream and ointment. Cream is preferable for treating exudative lesions, while ointment is preferable for dry lesions with a desquamative component (microbial eczema with lichenification). Fusidic acid and mupirocin are the most appropriate antibiotics; ideally they should be applied twice a day with bandage or 3 times a day without bandage for 7–10 days [63–65]. Since mupirocin-resistant staphylococci strains have been isolated, treatment should not be prolonged over 10 days [62]. Mupirocin should not be used in children younger than 1 year, because of a lack of studies on this age group. Since 2007, Retapamulin has been approved in the US for pediatric patients above 9 months old for the treatment of impetigo with S. pyogenes and methicillin-resistant S. aureus [66]. The recommended therapeutic regimen is 2 applications a day for 5 days [67]. The effectiveness of 5 days of Retapamulin ointment therapy is comparable to that of fusidic acid used longer; however it should be reserved for strains resistant to conventional treatments. The recurrence of infections in patients with AD is frequently associated with nasal colonization by S. aureus; when nasal swab is positive for S. aureas, nasal decolonization with mupirocin (after execution of antibiogram) has been proven effective, with 2 applications a day in both nostrils for 5 days per month, for a variable period of 3–18 months [63]. The most frequently reported side effects after the application of topical antibiotics include irritation, itching, and contact dermatitis. Episodes of contact dermatitis are generally attributed to excipients or preservatives contained in the medicine (i.e. lanolin, cetyl alcohol, stearyl alcohol) [68]. The existence of multiple strains of S. aureus resistant to the most common antibiotics represents a major challenge in the treatment of staphylococcal infections. Over the past 10 years, there has been a significant increase in impetigo caused by S. aureus resistant to treatment with fusidic acid, probably due to its widespread and often inappropriate use in chronic dermatoses [68, 69]. The use of topical antibiotics in the treatment of patients with AD and impetigo permits healing of the infection with less absorption and very low risk of systemic side effects. The duration of treatment should be restricted to the treatment of impetigo to prevent any risk of sensitization and/or the development of drug resistance.

### Summary box

- Topical antibiotics fusidic acid and mupirocin are indicated for the treatment of a monofocal bacterial superinfection (2–3 applications/day for 7-10 days).
- When S. aureus infection recurs, nasal colonization should be suspected and nasal swabbing should be performed. If positive, treat with nasal mupirocin for 3-18 months (2 applications/day for 5 days per month).
- For strains resistant to conventional treatments, retapamulin ointment is indicated as second-line treatment.

### Topical immunomodulators

Tacrolimus (Protopic®) and pimecrolimus (Elidel®) belong to the topical immunomodulators (TIMs) class that acts by inhibiting the activity of calcineurin; they were approved in 2000 and in 2001, respectively, for the treatment of AD in adults and children older than 2 years of age [70, 71]. Tacrolimus is produced by the bacterium *Streptomyces tsukubaensis,* while pimecrolimus is a chemical derivative of ascomycin, produced by *Streptomyces hygrospicus* [72]. TIMs inhibit the activation of T cells through a highly selective mechanism of action that provides good control of the disease and a low risk of side effects. They are especially useful when long-term therapy is needed or when TCS would cause undesirable side effects (eg. on sensitive areas like the eyelids) [73]. Tacrolimus also acts on eosinophils, basophils and mast cells by blocking the production of inflammatory cytokines and decreasing the activation of T lymphocytes by the Langerhans cells, while pimecrolimus also inhibits the release of inflammatory cytokines from mast cells [72]. TIMs are indicated in children older than 2 years suffering from mild/moderate AD (pimecrolimus) or moderate/severe AD (tacrolimus) [4] and meeting one of the following requirements: a) absence of response to the first-line therapy with topical corticosteroids; b) contraindications to treatment with topical corticosteroids; c) side effects induced by the use of topical corticosteroids, such as skin atrophy or telangiectasia; d) necessity of a long-term maintenance therapy [70]. Tacrolimus is available as in ointment: in children ages 2 to 15 years the 0.03 % formulation is indicated, while in patients ages 16 years and older the 0.1 % formulation is indicated. Pimecrolimus is available as a 1 % cream and has lower percutaneous absorption than the tacrolimus ointment. TIMs should be applied twice a day for 2–3 weeks, then once a day until resolution of the skin lesions and itch symptoms. If there isn’t any improvement after 2 weeks of treatment, alternative therapeutic options must be considered. The proactive therapy illustrated for TCS can be also adopted for TIMs; the patient’s clinical response should be re-evaluated after 12 months to decide whether to continue [4, 74]. If relapse occurs, the therapy can be restarted with two applications per day. Any lymphadenopathy should be noted before starting therapy and monitored throughout therapy [75]. The use of TIMs is contraindicated in patients with primary and/or acquired immunodeficiency, suspected bacterial or viral infection, eroded and/or exuding lesions, or significant sun exposure. Pimecrolimus and tacrolimus can cause an initial and transient burning sensation and/or itching at the site of application, especially if the eczema is in its acute phase; therefore patients should always be warned about the possibility of these effects in order to increase their adherence to treatment. Initial simultaneous treatment with corticosteroids can be considered to reduce the occurrence of burning or itching. In the literature, cases of allergic contact dermatitis and granulomatous rosacea-like reactions are reported as adverse effects following the implementation of TIMs. Labial melanosis may also be noted following the use of tacrolimus 0.1 % ointment [4, 76].

Several studies showed that intermittent or continuous use of TIMs did not cause systemic immunosuppression or increase the risk of bacterial or viral infections during five years of follow up [4, 77]. In 2006, the Food and Drug Administration reported the potential carcinogenicity of TIMs after a few reports of skin cancer and lymphoma in patients treated with TIMs. However, surveillance studies conducted on the drugs showed that the number of malignancies in patients with TIMs is lower than in the general population, and a surveillance study on the pediatric population showed that the prevalence of malignancies in children treated with TIMs is comparable to that in the general pediatric population [77, 78]. Monitoring of tacrolimus and pimecrolimus blood levels is not currently recommended for the topical treatment of AD [4].

### Summary box

- TIMs are a second-line therapy for AD.
- Tacrolimus ointment is used for moderate/severe forms:
  - 0.03 % formulation for patients aged 2–15 years;
  - 0.1 % formulation for patients ≥ 16 years.
- Pimecrolimus 1 % cream is indicated for mild/moderate AD in patients ≥ 2 years.
- Contraindications to the use of TIMs:
  - <2 years old;
  - congenital or acquired immunosuppression;
  - known or suspected infection;
  - eroded and/or exuding lesions
  - significant sun exposure.

### Wet-wrap dressing

Wet dressing, or wet-wrap therapy, consists of the application of a topical medication followed by bandaging with two layers of gauze or tubular dressings, the first of which is moistened and the second of which remains dry [79]. After a brief 5-min bath with warm water, the skin is patted dry, and then the topical medication is applied. Subsequently, the first layer of gauze is moistened with warm water, excess liquid is squeezed out, the moist gauze is applied to the skin, and then a second dry bandage is applied. If possible, the moistening of the first layer can be repeated every 2–3 h throughout the day after removing the dry layer. Steam can be used to moisten the first layer, but thorough cleaning of the vaporizer is recommended. The most suitable topicals for this treatment are fluticasone propionate, methylprednisolone aceponate, mometasone furoate, hydrocortisone acetate, or prednicarbate mixed with a hydrophilic emollient to 10 % dilution (1 part of steroid and 9 parts of emollient) for the body or a 5 % dilution for face [80–85]. Latex-free tight bandages and or elastic can be applied for 3 to 24 h at a time. Washable bandages can be used, but daily bandages are preferred. Wet-wrap dressing is a second-line treatment recommended for severe or refractory AD in patients older than 6 months of age [80, 81, 86]. No precise guidelines exist; effectiveness and the side effects are variable and depend on the age of the patient, the topical used, the time of occlusion, and the duration of treatment. The duration of treatment is variable from 2 to 14 days. The major results are obtained during the first week of treatment. Particular caution is necessary when applying TCS in patients at puberty, due to increased risk of developing striae distensae. The most dangerous side effect is systemic absorption of topical steroid, with transient increase in cortisol levels. Other possible side effects are folliculitis, bacterial or viral superinfection, and chills during the application of the wet layer if the water is not hot enough. Adherence and success can be increased through educational training [87].

### Summary box

- Wet dressing consists of a double layer of gauze or tubular dressings, the first of which is moistened, while the second layer remains dry.
- Wet wrap therapy is a short-term second-line therapy indicated for AD that is severe or resistant to topical treatments in patients older than 6 months of age.
- Topical steroids are used diluted with emollients to 10 % for the body and 5 % for the face.
- The most dangerous side effect is systemic absorption of steroid.

## Systemic therapies

### Systemic corticosteroids

Systemic corticosteroids have only a limited role in the therapeutic management of severe or difficult-to-control AD, both in children and adults [88–92]. Despite their anti-inflammatory effect, these agents do not act directly on the recovery of the skin barrier and can cause several significant side effects [88]. Often, a rebound effect, or relapse, occurs after their suspension. Studies supporting their effectiveness are few and the study populations are small. In fact, there is only one placebo-controlled study by Schmitt et al. [89] that evaluates the effectiveness of oral prednisolone and cyclosporine vs. placebo in patients who continued their topical therapy (emollients and steroids). Only 1 patient out of 27 taking systemic corticosteroids showed persistent remission (improvement > 75 % in baseline SCORAD after 2 weeks of oral steroids and 4 weeks of follow-up). This study was stopped for the appearance of significant eczema flares in the group treated with prednisolone. A recent review of systemic treatments for AD [90] identified only two other randomized controlled trials evaluating the efficacy of systemic corticosteroids in children. The first work evaluated the effectiveness of a 4-week therapy with beclomethasone dipropionate in 26 children with severe refractory eczema and found that the average SCORAD was reduced by 22 % without serious adverse effects [91]. In the second study [93], children were treated for two weeks with oral flunisolide, and eczema severity was reduced by an average of 39 % with no relapses during the 3 weeks following discontinuation. However, the quality of both of these studies is very low, and neither studied methylprednisolone, which is the corticosteroid most widely used in clinical practice. In an open study of 7 children with very serious unresponsive eczema, methylprednisolone was administered through IV bolus at the dose of 20 mg/kg/day for 3 days [94]. Skin lesions and itching improved for a few months without notable side effects apart from a significant but transient lymphopenia. The Practical Allergy (PRACTALL) Consensus Group guidelines [95] published in 2006 suggested that patients with acute flares may benefit from a short course of systemic corticosteroids, but their long-term use, especially in children, should be avoided. Therefore, the routine use of systemic corticosteroids in children is not recommended. Short courses of therapy may be offered in special situations, such as a severe exacerbation widely affecting the body surface with intense itching, a transition period before starting systemic non-steroidal immunomodulatory drugs, or in the presence of comorbidities such as a severe asthma exacerbation [96–98].

### Summary box

- The few available studies support a limited role for systemic corticosteroids in the management of severe AD in children.
- Exacerbation of symptoms often occurs after discontinuation of systemic steroids.
- Long-term use of systemic steroids may cause significant side effects.
- Short courses of therapy may be indicated in special situations.

### Systemic antimicrobials

Alteration of the skin barrier and defects of innate immunity predispose patients with AD to the complication of infections, mainly bacterial but also fungal and viral.

#### Bacterial infections

S. aureus, and secondly S. pyogenes, are the most common causes of bacterial superinfections (see Chapter 1.3 “Topical antimicrobials”). Over all, S. aureus can be isolated from up to 90 % of eczematous skin lesions. Colonization with S. aureas is less common in children younger than 2 years of age (50 % of lesions). The density of the bacterial colonies tends to increase with the clinical severity of atopic dermatitis [99]. However, treatment with systemic antibiotics should be reserved for clear evidence of bacterial infection [92, 97, 100–102]. The presence of bacterial colonies without clinical signs of infection does not warrant a systemic antibiotic [97, 103] (strength of recommendation B, level of evidence II [97]). Bacterial culture is not routinely required for antibiotic selection [104]. Uncomplicated bacterial infections, including those caused by staphylococci [105], can be treated with a beta-lactam antibiotic for 7–14 days. The first choice antibiotic is flucloxacillin (available only in tablet form in Italy); alternatives include amoxicillin and clavulanic acid or first- or second-generation cephalosporins such as cefuroxime and cefixime. Clindamycin (available only in tablet form in Italy) or macrolides can be used in subjects allergic to beta lactam; however, macrolide resistance is quite high [104]. If antibiotic therapy does not prove effective, compliance should be evaluated and cultures with antimicrobial sensitivity patterns should be obtained from both skin and nostrils. The diagnosis may need to be re-evaluated [4]. In patients hospitalized with severe infections not responsive to oral therapy, parenteral vancomycin, teicoplanin, or linezolid (second line) should be administered [105]. In case of recurrence, application of intranasal mupirocin twice a day for 10 days and/or bathing in water containing chlorhexidine or sodium hypochlorite solutions (0.005 %) are recommended to prevent or limit recurrences [106–109]. Other precautions for preventing infection include avoiding sharing toiletries, using liquid soap, and washing bedding weekly [107, 110].

#### Viral infections

Infection with HSV occurs in 3 % of patients and may cause systemic complications (eczema herpeticum, Kaposi’s varicelliform eruption) if not diagnosed and treated early. Generally, the infection is localized to the face and limbs, but it can be diffuse and accompanied by fever, lymphadenopathy, keratoconjunctivitis, or bacterial superinfection. Associated meningoencephalitis is uncommon [111]. Careful clinical observation is required to detect the characteristic clinical signs of umbilicated 2–4 mm diameter vesicles, which are often confluent and covered by serous, yellowish crusts [112]. Other conditions commonly included in the differential diagnosis include impetigo and more rarely “eczema coxsackium,” which does not require systemic treatment [113]. Children with eczema herpeticum require treatment with acyclovir as early as possible. The drug is typically administered orally but can be given intravenously if fever or systemic symptoms are present [104, 114, 115] (strength of recommendation C, level of evidence II [97]). Early use of this treatment has reduced mortality to zero [114, 116]. Treatment with calcineurin inhibitors is contraindicated during the acute phase [114]. Relapses occur in about 15 % of cases [117].

#### Fungal infections

The possibility of Malassezia restarting AD during adolescence [118, 119] via an immunological mechanism has been described in the literature. Malassezia may be implicated in AD affecting the head and neck and not responsive to the conventional therapy. In this subset of cases, oral treatment with itraconazole, ketoconazole or fluconazole has proven effective [120–122].

### Summary box

- Treatment with systemic antibiotics should be reserved exclusively for clear signs and symptoms of bacterial infection, not simple bacterial colonization.
- The first-choice antibiotic is a beta-lactam: flucloxacillin, amoxicillin and clavulanic acid or cephalosporins active on S. aureus, such as cefuroxime and cefixime.
- Additional treatment with intranasal mupirocin or baths with an antiseptic such as sodium hypochlorite can be useful to prevent or limit recurrence.
- Children with eczema herpeticum due to HSV require systemic therapy with acyclovir as soon as possible.
- In teenagers or others with AD localized to the head and neck that does not respond to therapy, the presence of Malassezia should be considered and an oral antifungal such as itraconazole may be administered.

### Systemic immunosuppressant agents

Systemic immunosuppressants represent a valid therapeutic option for severe, widespread and refractory forms of AD, since systemic corticosteroids have a limited role in the long-term therapeutic management [92]. Systemic immunosuppressants should be considered if the disease is having a significant negative impact on a child’s quality of life. However, therapy with systemic immunosuppresants should be restricted to specialized centers. There are few randomized controlled trials comparing systemic therapies, so assessing the relative efficacy of each agent is difficult. Most of the literature suggests the use of cyclosporin, but azathioprine and methotrexate are also recommended.

#### Cyclosporine

Cyclosporin A (CsA) is an immunosuppressive drug which acts directly on the immune system by inhibiting T-cell function [123, 124] and is indicated in the treatment of several skin diseases [125]. Its use in AD is based on evidence that AD results from the activation of a T-cell mediated inflammatory response of the skin (with a prevalence of Th2 response in the acute phase and Th1 in the chronic phase) [126]. The role of CsA in patients with severe AD that is refractory to conventional therapies is now confirmed (strength of recommendation B; level of evidence I-II [97]). The drug has shown considerable effectiveness in terms of reduction of clinical extension of the lesions, reduced itching and sleep deprivation scores, and improved quality of life [96, 127, 128]. However, 10 clinical trials have detected a rapid relapse of the disease within 8 weeks of discontinuation, with clinical scores returning to pre-treatment levels [129]. A recent study confirmed these findings, with a relapse rate of 42 %, especially in patients who had undergone short-course therapies [128]. CsA can be administered with high- or low-dose regimens and short- or long-term duration. Traditionally, an initial dosage of 5 mg/kg/day (up to 7 mg/kg/day according to some authors) is recommended for the first 2 weeks of treatment, followed by a maintenance dose of 1.5–3 mg/kg/day for a total period of 6–12 months [96]. Unfortunately, there is no evidence regarding the efficacy and safety of long-term therapy with CsA. However, in the short term, higher doses seem to be more effective in obtaining a rapid clinical response [130]. After an induction period, intermittent short-course therapy has been used to minimize risks without losing clinical benefits [131]. The side effects of CsA have led many physicians to advise regimens with the lowest effective dose for each patient [128, 130]. CsA has many reversible side effects, ranging from mild (headache, hypertrichosis, gingival hyperplasia, paresthesias), with a weekly rate between 1 and 28.2 % [96], to severe (hypertension, kidney failure), with a weekly rate between 0 and 2.2 % [130]. CsA treatment requires careful monitoring of blood pressure and renal function, and abnormalities warrant a reduction or suspension of therapy. However, several clinical trials have shown that the tolerability profile of this agent is better in children than in adults, with rare severe side effects in childhood [132–134]. Currently, there is no evidence of increased risk of lymphoproliferative diseases with prolonged CsA treatment of AD [127]. In conclusion, the current guidelines recommend CsA as the first-choice drug for severe AD refractory to topical first- and second-line treatments [112]. Nevertheless, caution is always advised in the pediatric age group, and short-term therapy is preferred [132].

#### Azathioprine

Azathioprine (AZA) is a purine analog which inhibits DNA synthesis, with preferential action on fast dividing cells, such as B and T cells during the inflammatory phase. Data suggests benefit in the pediatric population [135, 136], but the dosage and optimal duration of therapy still need to de defined (strength of recommendation B, level of evidence II [97]). The metabolism is of AZA is directed towards the activity of thiopurine-methyltransferase (TPMT), the main enzyme of thiopurine, of which different genetic polymorphisms affecting its action have been described. Myelotoxicity is associated with low TPMT activity [137]. The therapeutic range of AZA for AD is variable between 1 and 4 mg/kg daily [97], with 2.5 mg/kg daily being the most commonly used dose.

In Italy AZA is available only in 50 mg-tablets. Some patients may need prolonged treatment up to 12 weeks to obtain a full clinical remission. To monitor the possible adverse effects, TPMT activity (if available), peripheral blood counts, and liver enzymes should be measured before and during treatment [138]. Concomitant phototherapy is not recommended, due to increased risk of DNA damage and carcinogenesis, particularly with the exposure to UVA [138].

#### Methotrexate

Methotrexate (MTX) is an antifolate metabolite capable of blocking synthesis of DNA, RNA, and purines. It probably acts by inhibiting T-cell function. In recent years, several studies have highlighted the effectiveness of MTX in adult AD without serious side effects [139] (strength of recommendation B; level of evidence II [97]). Few controlled studies are currently available in pediatric age; thus, the use of MTX is mostly limited to clinical research [140, 141]. However, the use off-label of MTX can be considered in the absence of therapeutic alternatives [140]. The therapeutic dose in children is between 0.2 and 0.7 mg/kg/week, and the duration of therapy is of 10–16 weeks. Folate supplementation is always recommended during MTX treatment to reduce gastrointestinal and bone marrow toxicity. In an open randomized trial involving children with severe AD refractory to topical treatments and phototherapy, MTX and CsA had equivalent effectiveness, reducing SCORAD by 40 % after 12 weeks [141]. However, side effects are numerous. Blood cell count and liver and kidney function should be strictly monitored during treatment. Screening for hepatitis B and C as well as chest radiography should be performed before starting treatment.

**Biological drugs** (soluble receptors, monoclonal antibodies and cytokines such as recombinant interferon, anti-TNF, and inhibitors of the IgE/IL-5 pathway) are relatively new and lack sufficient data for recommendation in clinical practice.

### Summary box

- Use of immunosuppressant agents in AD should be restricted to specialized centers.
- CsA is an immunosuppressive drug recommended as first-choice therapy for severe AD refractory to first- and second-line topical treatments.
- CsA is effective in reducing clinical scores and improving children’s quality of life, but frequent relapse of AD after the end of treatment has been reported.
- Blood pressure and renal function must be carefully monitored during CsA therapy
- AZA and MTX may represent therapeutic options for severe and refractory AD, but controlled studies defining the optimal dosage and duration of therapy are still lacking.

### Antihistamines

Oral antihistamines (H1-receptor antagonists) block the effects of H1-receptor stimulation (vasodilation, erythema, edema) and have been included in the guidelines for AD treatment for a long time. However, their usefulness is controversial and debated [104, 142]. In general, the recent literature permits a short course in order to control itching. Some authors allow systemic therapy with H1 antihistamines only in patients whose AD is associated with symptoms of allergic rhino-conjunctivitis [97, 143, 144]. The pathogenesis of itch is very complex and related not only to the release of histamine [145], but also to the involvement of several other mediators such as proteases, gastrin-releasing peptide, substance P and IL-31. Experimental data show that the receptor PAR-2, present on keratinocytes and other skin cells and activated by proteases, plays a predominant role in mediating itch [146]. First-generation H1 antihistamines have lipophilic properties, which allow them to cross the blood–brain barrier and exert a subsequent sedative effect. Second-generation antihistamines have less lipophilicity and, therefore, do not cross the blood–brain barrier and are less sedating (Table 5). Due to the sedative properties of first-generation H1 antihistamines, both pediatricians and dermatologists allow short-term treatment with this medication to improve the patient’s quality of sleep [147]. On May 2014 the EMA observatory (European Medicines Agency) launched a review of drugs containing the antihistamine hydroxyzine because of possible adverse effects on the electrical conduction system of the heart [148]. In addition to H1 and H2 receptors, which are mainly located in the gastric mucosa, H3 receptors are expressed the central and (to a lesser degree) peripheral nervous system, and H4 receptors play a role in regulation of immune and inflammatory responses [149, 150]. The first results of the Early Treatment of the Atopic Child (ETAC) study [151], a multicenter trial on the efficacy of anthistamines (cetirizine/levocetirizine) to change the natural history of atopic diseases, published in 1998, showed good tolerability [152], but subsequent analysis of the benefit-risk-cost relationship was unfavorable, without no benefit in the prevention of asthma. Studies on the role of H4 receptors and their antagonists are ongoing, focusing on possible effects on the inflammatory response and, thus, on immune disorders, including AD The expression of H4 receptors on dendritic cells and keratinocytes favors a TH2-type response and the production of various mediators of inflammation, including IL-31, involved in the complex genesis of pruritus [153]. Finally, the topical use of H1 antihistamines is not recommended due to the risk of absorption and contact allergy [4].

**Table 5:** Classification of antihistamines (from AIFA repository 2015)

First-generation H1 antihistamines	Second-generation H1 antihistamines	Active metabolites of second-generation H1 antihistamines (third- generation H1 antihistamines)
Alkilamines: Chlorpheniramine	Cetirizine	Levocetirizine
Dimethindene	Loratadine	Decarboetoxiloratadine or Desloratadine
Ethanolamines: Diphenhydramine	Ebastine	Norastemizole
Ethylenediamines: Thonzylamine	Acrivastine	Fexofenadine
Phenothiazines: Promethazine	Astemizole	
Piperazines: Hydroxyzine	Terfenadine	
Piperidines: Ciproeptadina	Azelastine	
Oxatomide	Mizolastine	
*Intermediate degree of lipophilicity*	Bilastine	
Ketotifen	Rupatadine	
	Levocabastine	

### Summary box

- There is no evidence to support widespread use of antihistamines in AD.
- Short-term and intermittent courses of sedating (first-generation) antihistamines can be used in children if itch strongly affects quality of sleep.
- Topical use of antihistamines is not recommended due to risk of absorption and contact allergy.

### Phototherapy

Phototherapy is a second-line treatment, used when the AD is not controlled with emollients, topical steroids and TIMS [92, 154]. Numerous studies have demonstrated the efficacy of phototherapy in AD, both in the acute and the chronic phase [97, 155]. Currently available devices emit spectra of UVA and UVB radiation, especially narrow-band UVB (UVBTL01 311–313 nm) and UVA1 (340–400 nm). Phototherapy with UVA1 can be carried out with high (130 J cm2), medium (50 J cm2) or low -dose (10 J cm2). The mechanism of action of UV in AD is not completely understood, but an antimicrobial effect that reduces colonization of *S. aureus* and induces cytokine production with anti-inflammatory and immunosuppressive properties is assumed. Phototherapy with UVBTL01 is indicated in moderate forms of chronic AD, while high-dose UVA1 is preferred for severe and exuding AD. Phototherapy with UVBTL01 is most widely used due to availability, tolerability, effectiveness, and low carcinogenic risk in long-term use [155, 156]. However, it is not indicated in children younger than six years. To date, no clear guidelines for phototherapy in AD are available, and patients typically follow protocols for psoriasis (2–3 sessions per week). The starting dose is calculated based on the minimum erythema dose or Fitzpatrick skin phototype. Emollients may improve the effectiveness of treatment and topical steroids can be used to better control eczema before starting the phototherapy. Data on the duration of remission and comparing various approaches are missing. Common side effects at the beginning of the treatment include itching, burning, erythema, and folliculitis. The long-term risk of UVBTL01 phototherapy is unknown; nevertheless, its safety profile has been proven in patients with psoriasis.

### Summary box

- Phototherapy is a second-line treatment to be used when the AD is not controlled by other therapies.
- Narrow-band UVB (UVBTL01) is most commonly used and is indicated for children six years of age and older.
- Psoriasis treatment regimens are followed, with 2–3 sessions per week for a duration of a few months, depending on clinical response.
- Long-term carcinogenic effects have not been reported.

### Allergen immunotherapy

Theoretically, allergen immunotherapy (AIT) may represent an additional therapeutic option for a subgroup of patients in which interventions of environmental prevention against mites (discussed below) has not led to significant improvement. The principle of AIT is administering increasing doses of clinically relevant allergen(s) in order to induce immunological tolerance in a sensitized patient [157, 158]. The therapeutic efficacy of AIT has been clearly demonstrated in allergic asthma, rhinitis and hymenoptera venom allergy [159–162]. Despite this, AIT has not been shown to be an effective treatment for AD. In fact, while the 2012 European guidelines suggested a potential benefit of AIT in a select group of patients with coexistent allergic sensitization and AD sensitization, the latest AAD guidelines published in 2014 do not mention this therapeutic option [97].

For many years, AIT had not been considered in the treatment of AD, since it was believed to be associated with a worsening of the disease. However, the latest studies have found good tolerability of the treatment in most of patients [163–166]. Since 1974, several clinical studies on the treatment of AD with AIT have been performed, some of which included pediatric subjects (Table 6). Otherwise, few controlled studies are available. Authors of recent reviews conclude that there is no evidence to recommend the use of AIT in the treatment of AD [167, 168]. However, some authors identify as potential candidates for AIT a subgroup of highly selected patients with the following characteristics: a) IgE-mediated sensitization (in particular to house dust mites, but also birch and grass pollens); b) severe AD (SCORAD > 50); c) Atopy Patch Test positive for the eliciting allergen. A recent meta-analysis of 8 RCTs including adult and pediatric patients found that AIT was effective, but subgroup analysis of the four studies with exclusively pediatric populations did not show significant benefits [164]. Analysis of the safety profile of the treatment found no significant difference between patients and controls, and no severe adverse reactions were reported in any of the included studies. Although the meta-analysis favored the efficacy of AIT for AD, the validity of meta-analysis’s methodology has been questioned [169]. Randomized clinical trials and observational long-term placebo-controlled studies evaluating the “disease modifying” effects of this treatment are lacking. Therefore, its therapeutic indication in patients affected exclusively by AD remains controversial [3].

**Table 6:** Observational studies and randomized controlled trials (RCTs) on allergen immunotherapy (AIT) which included pediatric patients with atopic dermatitis (AD)

Study	Study design	Patients n (age, yrs)	Allergen	Route	Duration (months)	Outcome	Adverse reactions % AIT compared to control group	Efficacy
Kaufman et al. 1974 [271]	qRCT DBPC	52 (2–47)	Danders	SCIT	24	Success of the treatment (Physician)	Systemic: NA	Positive in 81 % of treatment group vs. 40 % of controls
			HDM					
			Moulds				Locals: 50 vs 40	
			Pollens					
Warner et al. 1978 [272]	RCT DBPC	20 (5–14)	HDM	SCIT	12	Success of the treatment (Patient)	Systemic: NA	Positive
							Locals: NA	
De Prisco de Fuenmayor et al. 1979 [273]	Obs.	15 (6–14)	Airborne allergens	SCIT	-	Clinical assessment	Occasional exacerbation of AD	Positive in 60 % of patients
Ring 1982 [274]	PC	2 twins (10)	Pollens (grasses)	SCIT	24	Clinical assessment sIgE (grasses) Total IgE	Occasional exacerbations of eczema in SCIT patients	Positive
Seidenari et al. 1986 [275]	Open	63 (4–45)		SCIT	6–24	Clinical assessment	Occasional mild exacerbation	Positive in 65 % of patients
Glover et al. 1992 [276]	RCT DBPC	24 (5–16)	HDM	SCIT	8 (phase I)	Success of the treatment	Systemic: 0 vs 8	Uncertain with a possible positive effect of prolonged treatment (phase II)
					6 (phase II)	(Patient)	Locals: NA	
Heijer et al. 1993 [277]	Obs.	93 (6–66)	Airborne allergens	SCIT	39	Clinical assessment	Asthma, RC, fever, fatigue, itching, dizziness	Positive
						Total IgE		
Galli et al. 1994 [278]	RCT PC	34 (0.5–12)	HDM	SLIT	36	Success of the treatment (Physician)	Systemic:0	Negative
							Locals: 6.3 vs 5.6	
Trofimowicz et al. 1995 [279]	Obs.	22	HDM	SCIT	36	Clinical assessment	-	Positive in 75 % of patients treated with AIT for HDM and up to 80 % of patients treated with AIT for pollens
			Pollens					
Zwacka et al. 1996 [280]	Controlled	212 (6–15)	Airborne allergens	SCIT vs SLIT	24	Clinical assessment	-	Positive (both SLIT and SCIT)
						Total IgE		
Czarnecka-Operacz et al. 2006 [281]	RCT DBPC	66 (5–44)	HDM	SCIT	48	Success of the treatment (Physician)	-	Positive
		- 37 AIT	Pollens					
		-29 Controls						
Pajno et al. 2007 [282]	RCT DBPC	56 (5–16)	HDM	SLIT	18	SCORAD (Physician)	- Local reactions in 7 SLIT patients	Positive only in patients with mild to moderate AD, not in those with severe
							- Itching and erythema in 2 SLIT patients	
		-28 AIT				Pharmacotherapy		
		-28 Controls						
Cadario et al. 2007 [283]	Obs.	86 (3–60)	HDM	SLIT	12 (at least)	SCORAD	No severe reactions	Positive
						Total IgE and sIgE		
Bussmann et al*.* 2007 [284]	Obs.	25 (5–65)	HDM (allergoid)	SCIT	7	SCORAD	-	Positive
						sIgE; sIgG4		
						IL/mediators		
Nahm et al. 2008 [285]	Obs.	20 (7–58)	HDM	SCIT	12	Clinical assessment	None relevant	Positive
						SCORAD		
Kwon et al. 2010 [286]	Obs.	20 (6–33)	HDM	SCIT	12–60	Clinical assessment	No exacerbations	Positive
						sIgE (DP)		
						Total IgE		
						Chemokines		

*qRCT* quasi-randomized controlled trial, *DBPC* double blind placebo controlled, *HDM* house dust mite, *Obs.* observational, *PC* placebo controlled, *SCIT* subcutaneous immunotherapy, *SLIT* sublingual immunotherapy, *DP Dermatophagoides pteronyssinus*

### Summary box

- Although its safety profile is favorable, AIT is not an appropriate therapeutic option for all patients suffering from AD.
- Its indication in clinical practice is limited by the small number of controlled randomized clinical trials and the high heterogeneity of the published works.
- AIT is most strongly indicated in a sub-group of patients with the following characteristics: sensitization to dust mites, clear correlation between allergen exposure and clinical manifestations, and severe AD.

## Non-pharmacological interventions

### The role of foods

The link between AD and food allergy (FA), including FA’s possible role in causing AD, remains controversial [170]. Support for the causal link comes from the observation of immediate-type allergic reactions in children with AD after the elimination and subsequent reintroduction of certain foods, especially cow’s milk (CM) and hen’s egg [171–176]. Additionally, many studies have shown that the more severe the AD [176–179] and earlier its first appearance [180], the greater the association with IgE-mediated FA comorbidity. The foods that most often cause FA in patients with AD are CM, egg, peanut, wheat, soy, nuts, and fish [179].

Although AD has proved to be a very complex and heterogeneous disease, it is possible to identify two main subtypes based on whether IgE levels are elevated (IgE-associated/extrinsic AD) or normal (non-IgE-associated/intrinsic AD). The latter, which is more common in preschool-age children and adults [181, 182], is associated with a lesser risk of developing asthma. According to Bergmann et al. [183], the patterns of clinical reactions to food in patients with AD can be divided into three groups: a) immediate-type reactions, usually IgE-dependent, developing within 2 h and characterized by urticaria, angioedema, rash, itching and possible involvement gastrointestinal and respiratory tract; b) delayed-type reactions that occur 6 to 48 h after the food introduction, with typical areas of eczema and are typically non-IgE mediated; c) mixed type, occuring in 40 % of children with AD and positive food challenge [184]. In 1978 a double blind controlled crossover study by Atherton et al. [185] first demonstrated an improvement in AD following a diet without CM and egg. Subsequent studies on patients with AD observed only immediate symptoms after consumption of trigger foods, suggesting a comorbid FA [172, 174, 176, 186]. Rowlands et al. [187] were able to demonstrate a link between reintroduction of previously eliminated foods and late recrudescence of AD only in one out of 17 children hospitalized with severe AD. Other studies showing the onset of late eczematous-type reactions not preceded by immediate allergic reactions are scarce and of variable quality [175, 188]. The key question is whether food allergy in the context of IgE-associated AD is an unrelated condition or whether it can trigger or worsen AD. Another option is that the food directly causes a delayed reaction. Rowlands et al. [187] assert that although it is possible that the food might induce a true eczematous lesion, this is the exception rather than the rule.

Genetic predisposition that leads to abnormalities of the skin barrier has been accompanied by an abnormal immune response that makes the skin even more vulnerable to environmental factors. Individuals with AD and concomitant filaggrin (FLG) deficiency tend to have earlier onset of the disease, which in turn is more severe and persistent and more commonly associated with allergic sensitization [189–191]. It should however be noted that all patients with AD exhibit a barrier defect, but not all patients with FLG deficiency have AD.

In particular, a link has been observed between FLG gene mutation and peanut allergy [192]: the FLG deficiency allows food allergens to penetrate through the skin, interact with the immune system, and induce FA. In fact, although the sensitization to food classically happens through the intestines, Du Toit et al. [193] have shown that peanut sensitization can occur without previous ingestion, by simply applying peanut dissolved in oil to inflamed skin, and that early introduction decreases the frequency of peanut allergy. Also, Fox et al. [194] have found an association between environmental exposure to peanut and risk of developing peanut allergy; trace amounts of peanut can be found in furnishings or on hands [195].

The above data recently has led to the suggestion to overturn the pathogenic mechanism linking FA to AD: as opposed to FA inducing AD, exposure of skin with a barrier defect to a food may cause first a sensitization and then, later, a real FA [196–198]. By contrast, the early consumption of food would induce, through the gastrointestinal system, a food tolerance [199–201]. The timing and the balance of dermal and/or intestinal exposure would determine whether the child develops a food allergy or a food tolerance [202]. This mechanism has been confirmed in some mouse models, where the abrasion of the skin and subsequent deposition of peanut or ovalbumin leads to a significant specific IgE response [203, 204]; this state, in turn, can result in a clinically evident FA, as is the case with AD patients whose skin is exposed to peanut oil [193], especially if allergen doses are high. This new view of the problem could be simplistic in that it attempts to export the peanut model to other foods, in particular in CM, egg and wheat. However, it is likely that the concentration in the air of food allergens other than peanut is not as high as the concentration of peanut and inhalant allergens.

Additionally, in AD patients, the dynamics of sensitization to food allergens varies from food to food [205]; peanut acts similarly to an inhalant allergen, with a continuous increase in the concentration of specific IgE over time, while CM- and egg-specific IgE tend to decrease over time. Furthermore, the concentration of food allergens present in mattress dust varies from food to food [206]. Finally, it is known that children with AD may be sensitized to egg [207] without ever having ingested it [208]. The above is to emphasize that although the skin of the subjects with AD may allow the entry of food allergens, it is not the only point of entry for food allergens.

FA is also seen in people without AD or other skin damage [209]. In the study by Flohr et al. [210] of 619 exclusively breastfed children at 3 months of age, 154 (24.9 %) suffered from AD, but only 24 % of the babies with AD had a FLG mutation (vs. 8.4 % of those without AD). AD was found to significantly correlate with food sensitization but not with the FLG mutation [210]. Flohr et al. argue that since the children were breastfed and there are few allergens in breast milk, sensitization had occurred through the skin, not through the intestines. This argument is consistent with the previous demonstration of peanut sensitization in AD patients by application of peanut oil to the skin [193]. In our opinion, this explanation seems forced since detection of considerable quantities of heterologous proteins has been widely demonstrated in breast milk from mothers of both infants with AD as well as healthy infants [211]. It would then require individual predisposition or specific intestinal conditions to ensure that a protein first induced sensitization and then caused a real FA.

In sum, lesional skin is one of the possible routes of entry for allergens, as the direct contact of lesional skin with peanuts and/or other foods (directly spread on the skin or through contact with contaminated hands) may facilitate the occurence of sensitization. Finally, it must be considered that allergic inflammation of any type may damage the same skin barrier and inhibit the formation of FLG [212].

### Summary box

- AD is a multigenic and multifactorial disease often associated with increased production of total and/or specific IgE. This may be associated with sensitization to foods, inhalant allergens, or both.
- Allergic sensitization may occur through the gastrointestinal (breast milk) or the transcutaneous route.
- Allergic inflammation can alter the skin barrier by reducing the production of FLG.
- If a child with AD is suspected of having a FA, assess whether there is a food sensitization and whether symptoms (shock, urticaria, persistent diarrhea, asthma) require an elimination diet. Generally, it is preferable not to eliminate foods in sensitized patients without relevant immediate reactions.
- In subjects with moderate-severe AD, skin testing, particularly for the egg, may be justified. If positive, egg should be introduced in a protected environment, for the risk of immediate allergic reactions.
- In the event that appropriate therapy with emollients, topical corticosteroids and/or systemic treatments is ineffective and there is suspicion for a food a trigger, a short elimination diet and subsequent food challenge may be attempted.
- However, some advise against attempting elimination/reintroduction diets due to the possibility that reintroduction of the suspect food could cause a severe allergic reaction.

### Role of the environmental triggers

Several environmental factors seem to play a role in AD, particularly in exacerbations. In addition to mechanical and chemical irritants (e.g. wool, irritant soaps, toiletries containing alcohol or perfumes), indoor allergens, environmental pollutants, and climatic changes may play a role.

#### House dust mites

House dust mites, *D. pteronyssinus* and *D. farinae*, have been recognized as the main source of household allergens, especially in temperate climates [3, 213].

Mites release several allergens into the environment, including cysteine-protease (Der p 1, Der f 1), serine proteases (Der p 3, 6 and 9), glycosidases, carbohydrate-binding proteins, calcium-binding proteins, and cytoskeletal and muscle proteins [214]. In addition to the classic route of allergic sensitization, there is evidence that some allergens (such as proteases) can also exert a pro-inflammatory non-IgE mediated activity directly on the epithelial barrier, both stimulating receptors of the innate immune system and releasing pro-inflammatory cytokines and interleukins [214–216].

Several studies have also observed a correlation between the degree of exposure to mite allergens during early childhood and risk of allergic sensitization [217–219]. In some patients with AD, it has been documented that exposure to dust mites by direct contact or inhalation can exacerbate the disease [220]. However, the role of this exposure in the pathogenesis of AD remains controversial, since positive correlations were mainly in observational studies [221]. In addition, studies assessing the effectiveness of prophylactic anti-mite environmental interventions (e.g. anti-dust mite covers), showed reduction in the concentration of dust mite allergens in domestic areas but did not show reduced risk of allergic sensitization or reduced AD severity [222–224]. However in some cases, especially in patients with more severe AD, a significant improvement in clinical AD scores due to stringent prophylactic environmental measures, was shown [225]. Similar but less significant results have been observed in two additional studies, one of a pediatric population with AD and the other of an adult population with AD [226, 227]. Studies on the relationship between AD and the environment have not yet confirmed the precise role of environmental dust mites in AD. However, one of the possible reasons for discrepancies is the lack of homogeneity in the prophylactic measures used [228–230]. Currently, recommendations for mite prophylaxis suggest the simultaneous application of multiple interventions, such as reducing the relative humidity in the house (the main risk factor for mite growth) to around 50 %, using anti-mite covers, eliminating the main sources of allergen accumulation (e.g. carpets, rugs, curtains, plush), washing pillowcases weekly at high temperatures (about 60 °C), and vacuuming with a machine containing a HEPA filter capable of removing allergens [228]. The American Academy of Dermatology recently recommended considering anti-mite prophylactic measures (especially anti-mite pillowcases and mattress covers) for AD patients sensitized to dust mites and insufficiently controlled by topical therapy [97].

#### Pets

In addition to mites, pet allergens have been proposed to play a role in AD. However, at the present, the relationship between early-life exposure to these allergens and the appearance of allergic sensitization and allergic diseases is still controversial. Recent cohort studies indicate that the first year of life is a critical period, during which exposure to pets, especially dogs, may reduce the risk of developing allergic sensitization and AD later in life [231]. The results are not homogeneous and are complicated by confounding factors, such as family history of atopy and exposure to dog and cat allergens outside the home [232]. Therefore, at present no guidance exists about keeping or removing pets already living in the house in order to prevent development of allergic diseases including AD [233].

#### Environmental pollutants

Increasing evidence shows a correlation between the level of environmental pollutants and respiratory allergic diseases, especially in regions undergoing rapid industrialization [234]. However the role of exposure to such pollutants in AD has not been defined, as only few prospective studies have documented an association between AD and concentrations of pollutants, especially those produced by vehicular traffic (nitrogen oxide, particulate matter [PM]) [235]. However, recent evidence seems to support the role of environmental pollutants in exacerbating AD. Higher concentrations of PM 10 and volatile organic compounds have been linked with AD exacerbations [236], while clinical improvement was observed with increased cleaning in kindergarten classrooms and reduced environmental PM 10 [237].

#### Climatic influences

Recent attention has been paid to the role of climatic variations, which may affect differences in the prevalence of AD worldwide. Osborne et al*.* have documented a lower prevalence of AD in Australian children living in regions closer to the equator [238]. Similarly, Silverberg et al*.* have shown that the prevalence of AD in the United States is lower in the regions with the highest solar exposure [239]. These data support the hypothesis of a link between AD and levels of vitamin D. On the other hand, a recent prospective study observed that greater sun exposure and elevated temperatures were associated with uncontrolled AD [240, 241]. Although such correlations do not prove a causal effect, it is necessary to educate the patient and family about the possible role of climatic factors, with particular attention to exposure to very high temperatures (both environmental and household during the winter season), which can increase sweating and water evaporation from the skin, exacerbating dryness and skin irritation [240].

### Summary box

- Avoid mechanical and chemical irritants (eg. wool, irritant soaps, toiletries containing alcohol or perfumes).
- Anti-mite prophylactic measures should be recommended for AD patients sensitized to mites and insufficiently controlled by topical therapy.
- There is no conclusive evidence to recommend keeping or removing household pets for the sake of preventing sensitization.
- Emollient therapy should be adapted to climactic characteristics (relative humidity, solar exposure, temperature).

### Textiles

In patients with AD, direct contact with certain textiles with rigid fibers (e.g. wool or nylon) is a source of irritation [242], while the use of soft fabrics (e.g. knitted silk; cotton, with or without silver enrichment) may reduce the skin irritation [3, 243]. Soft fabrics reduce friction, have transpiring properties and allow the absorption of sweat and exudates, which are important in maintaining the hydrolipidic film of the skin [244]. The effectiveness of Dermasilk®, a new knitted silk fabric with antimicrobial properties, has been evaluated in a very limited number of controlled clinical trials. The studies have shown encouraging results, with improvement of clinical scores and reduction of disease recurrence during the maintenance phase [244–247]. In a study of Gauger et al*.* [248], tissues enriched with silver also were effective in improving AD symptoms within 2 weeks of use. However, the risk of percutaneous absorption of silver should be always considered, especially in damaged skin [249]. ZnO-functionalized texile fibers (BenevitZink+) also seem to improve AD severity [250]. In view of the limited number of clinical studies carried out and the high cost of these materials, the most recent American guidelines conclude that there is limited evidence to support the use of these textile products in treatment of AD [97]. In any case, direct contact between irritating fabrics or wool and the skin should be avoided.

### Summary box

- Avoiding contact with irritating fabrics is essential AD patients.
- The use of textile products with antibacterial action must be confirmed by further studies.

### Balneotherapy

Balneotherapy is the immersion of the body or parts of it in bathtubs or pools of mineral water. Mineral waters may be categorized as sulphurous, bicarbonate, sulphate, carbonic, arsenical and ferruginous [251] and as hypotonic, isotonic or hypertonic. Temperature may vary between 20 and 40 °C. To be considered “medical,” the water must have a minimum concentration of ion and/or gas to induce relevant clinical effects. The composition and threshold dose of the thermal water may vary from one region to another and among health resorts [252]. Among the mineral waters, calcium-bicarbonate magnesium water is most indicated for AD treatment. For many years, Dead Sea waters have been used in the management of skin disease, since they are the salitiest on earth (salt content is 350 g/l vs. 40 g/l for most oceans or seas [253]) and particularly rich in salts such as MgCl2, CaCl2, KCl, and MgBr2. The presence the CaCl2 gives to the Dead Sea waters [254] a slimy feeling. The Dead Sea waters are very rich in magnesium, which promotes cell maturation and keratinocyte differentiation and is particularly useful in patients with psoriasis [255]. It is also a natural moisturizer that can improve the skin’s capability to retain water. Retrospective studies have shown the benefit of the climate and the Dead Sea salts for patients suffering from AD and other skin diseases, in the absence of significant side effects [256]. Unfortunately, few studies have been published, and they have been methodologically insufficient. The beneficial effects of treatment with Avène water for 3 weeks were evaluated in a observational study [257]; patients bathed for 20 min at 32 °C and showered to remove scales 6 days out of 7 for three consecutive weeks. At the end of the therapy, the clinical score of the AD improved significantly with long-term benefits. An open randomized controlled trial comparing the efficacy of the combination of baths of duration ≥ 4 min in a solution of 10 % Dead Sea salts plus phototherapy (exposure to 15–30 min to UVB 311 nm) vs. phototherapy alone detected greater clinical improvement in the group that bathed with the Dead Sea salts [258]. Finally, an open randomized controlled trial [259] evaluated the efficacy of the hot springs baths of Terme di Comano (Trento, Italy) in 104 children between 1 and 14 years with mild to moderate AD. Patients were alternately assigned to balneotherapy or to topical corticosteroid treatment for 2 weeks. At the end of the study, significant improvement in eczema severity and the quality of life was seen in both groups. The group treated with corticosteroids experienced more pronounced initial clinical improvement, but after 4 months, the group receiving hydrotherapy had significantly fewer and shorter exacerbations. The effects of hydrotherapy are enhanced by coexisting factors such as the relaxing spa environment [241], climatotherapy (warm weather), sun exposure (light therapy), and therapeutic education program [260]. Balneotherapy is generally not recommended during acute eczema, especially in children. Contraindications are febrile or exanthematous diseases and impetiginization. After 5–7 days of treatment, a transient exacerbation of the disease (“spa reaction”) can occur; this phenomenon is most likely to be observed if the dermatitis is not well controlled, and it usually resolves at the end of the treatment [259]. In conclusion, balneotherapy is a possible adjuvant therapy in the management of AD, but further studies are necessary to confirm its efficacy.

### Summary box

- Balneotherapy is possible adjuvant therapy in the long-term management of the AD.
- Further randomized controlled studies are needed to assess its effectiveness.
- The effects of hydrotherapy are probably enhanced by other factors such as the relaxing spa environment, the warm climate, increased sun exposure and therapeutic education program.

### Educational interventions

The complexities of AD can be summarized as follows: 1) multifactorial etiopathogenesis; 2) chronicity; 3) early age of onset; 4) involvement of the family system.

Both genetic and environmental factors contribute to its complexity. The latter are considered an expression both of the physical environment (such as health and climate) and the psycho-social system. Chronicity requires that patients (depending on age) and their caregivers adequately understand the disease and the required treatment and comply with the programmed treatment [261]. The early age of onset (0–14 years), and the varying clinical manifestations of AD at different stages of development, require physician knowledge of the psycho-social and relational implications of the disease so that interventions may be adapted accordingly. The family is a dynamic system that is critical in the emotional-affective and educational development and psychological well being of the patient. A family dealing with a chronic disease, such as AD, requires multidisciplinary support which can be summarized in the following three levels: 1) bio-pharmacological; 2) educational, pedagogical and instructive; 3) psychological/psychotherapeutical. In fact, in recent years, the scientific research on the treatment of AD has shifted from the biomedical, technical and “paternalistic” model toward a bio-psycho-social and educational one.

**Therapeutic education**, as defined by the WHO [262], allows us to provide not only technical information about the disease and corresponding treatments but also a customized plan developed in partnership with the patient and the patient’s caregivers.

It is important to consider how emotional factors like insecurity, inadequacy, anxiety, and depression affect atopic patients [263]. Additionally, the family is afflicted by both economic stress related to the cost of treatment and by a significant psychological burden, which is particularly heavy in young and severely affected children [264]. These factors may affect treatment compliance.

The **Ideal Model of Intervention** integrates different theoretical and operational models, with participation of a multidisciplinary team composed of the specialist physician (Pediatrician, Allergist, Dermatologist), the psychologist/psychotherapist, and other professionals, such as nurses.

The specialist acts as the expert in therapeutic education and first conducts a psychologically supportive educational interview, covering:

- questions about the onset, history and evolution of the disease, as well as challenges in adhering to treatment
- evaluation of symptoms, such itching and sleep disturbance, by quantitative tools, such as the Patient Oriented SCORAD (PO-SCORAD) [265] and a Visual Analogue Scale of Pruritus;
- strategies for coping with itching and sleep disturbances [266]

Discussing challenges with treatment compliance, itching, and sleep allows for re-education about more effective strategies and improves adoption of these strategies.

A second education-oriented interview can be conducted by the broader team of physicians (dermatologist, pediatrician, allergist), nurses and psychologist with specific objectives:

- teaching the recognition of skin lesions and the application of topical medications, overcoming corticophobia, preventing flare-ups
- demonstration by nurses of techniques for bathing, applying topical medications while performing baby massage, and bandaging. The aim is for bathing, moisturizing, and dressing to be positive occasions of play.

These sessions can be conducted as large group demonstrations or, preferably, as smaller workshops, with more opportunity for practical individualized demonstrations [267].

After this phase, a *psycho-diagnostic step* may be offered for the patient and parents if the family requests it or if the staff believes it is indicated [268].

**Psychotherapeutic Intervention** for the patient is necessary when individual/family psychopathological suffering is severe, as may be the case in severe AD. The clinical assessment includes some psycho-diagnostic testing for parents and patients over the age of 4 years, which helps identify the most useful psychotherapeutic intervention [269]. Psychotherapeutic intervention, provided by a professional expert, supports the patient and family in coping with the emotional pain associated with the disease, improves the stability of their existential and social life, and ultimately improves adherence to treatment.

In conclusion, the paradigm of therapeutic education is the basic element of the AD Intervention Model (Fig. 1). It considers the relationship with the patient-family the essential part of the multifactorial approach, in which the clinical team works as a whole. When a team approach is not feasible, this model can still be used as a theoretical guide for the specialist, even if in a simplified form.

### Summary box

- The complexity of care for AD can be summarized in the following three levels: 1) bio-pharmacological; 2) educational, pedagogical and instructive; 3) psychological/psychotherapeutical
- Therapeutic education allows us to provide not only technical information about the disease and corresponding treatments, but also a customized treatment plan developed in partnership with the people involved in the case.
- The Intervention model requires a multidisciplinary team composed of the specialist physician (pediatrician, allergist, dermatologist), psychologist/psychotherapist, and other professionals including nurses, oriented toward improving not only the disease but also the quality of life of children and their families.

## Conclusion

The Italian Consensus Conference on clinical management of atopic dermatitis in children integrates the basic principles of the most recent guidelines for the management of atopic dermatitis to facilitate a practical approach to the disease. Levels and models of intervention are also enriched by the Italian experience to facilitate a practical approach to the disease. The therapeutical strategy, and in particular the selection of therapies and the application of topical products should be adapted to the clinical severity and require a tailored strategy to ensure good compliance by children and their parents.

## Abbreviations

AD: Atopic dermatitis
AIT: Allergen immunotherapy
AZA: Azathioprine
CM: Cow’s milk
CsA: Cyclosporin A
FLG: Filaggrin
HSV: Herpes simplex virus
MTX: Methotrexate
NMF: Natural moisturizing factor
RCTs: Randomized controlled trials
TCS: Topical corticosteroids
TIMs: Topical immunomodulators
TPMT: Thiopurine-methyltransferase
